# Observational study on the impact of initiating tiotropium alone versus tiotropium with fluticasone propionate/salmeterol combination therapy on outcomes and costs in chronic obstructive pulmonary disease

**DOI:** 10.1186/1465-9921-13-15

**Published:** 2012-02-17

**Authors:** Arjun Chatterjee, Manan Shah, Anna O D'Souza, Benno Bechtel, Glenn Crater, Anand A Dalal

**Affiliations:** 1 Department of Internal Medicine, Wake Forest University School of Medicine; Wake Forest University School of Medicine, Medical Center Blvd., Winston-Salem, NC 27157, USA; 2 Data Analytics and Insights, Xcenda; 4114 Woodlands Parkway, Suite 500, Palm Harbor, FL 34685, USA; 3 US Health Outcomes, GlaxoSmithKline; 5 Moore Dr, Bide West, Mail Stop B.3153, Research Triangle Park, NC 27709, USA

**Keywords:** chronic obstructive pulmonary disease, pharmacoeconomics, cost, hospitalization, emergency room visit, pharmacotherapy, exacerbation, add-on therapy, triple therapy

## Abstract

**Background:**

This retrospective cohort study compared the risks of exacerbations and COPD-related healthcare costs between patients with chronic obstructive pulmonary disease (COPD) initiating tiotropium (TIO) alone and patients initiating triple therapy with fluticasone-salmeterol combination (FSC) added to TIO.

**Methods:**

Managed-care enrollees who had an index event of ≥ 1 pharmacy claim for TIO during the study period (January 1, 2003-April 30, 2008) and met other eligibility criteria were categorized into one of two cohorts depending on their medication use. Patients in the TIO+FSC cohort had combination therapy with TIO and FSC, defined as having an FSC claim on the same date as the TIO claim. Patients in the TIO cohort had no such FSC use. The risks of COPD exacerbations and healthcare costs were compared between cohorts during 1 year of follow-up.

**Results:**

The sample comprised 3333 patients (*n *= 852 TIO+FSC cohort, *n *= 2481 TIO cohort). Triple therapy with FSC added to TIO compared with TIO monotherapy was associated with significant reductions in the adjusted risks of moderate exacerbation (hazard ratio 0.772; 95% confidence interval [CI] 0.641, 0.930) and any exacerbation (hazard ratio 0.763; 95% CI 0.646, 0.949) and a nonsignificant reduction in COPD-related adjusted monthly medical costs.

**Conclusions:**

Triple therapy with FSC added to TIO compared with TIO monotherapy was associated with significant reductions in the adjusted risks of moderate exacerbation and any exacerbation over a follow-up period of up to 1 year. These improvements were gained with triple therapy at roughly equal cost of that of TIO alone.

## Background

The goals of pharmacologic therapy for chronic obstructive pulmonary disease (COPD)-to control symptoms, improve health status, and reduce the frequency of exacerbations [1]-cannot be met with monotherapy for many patients. Guidelines on COPD management recommend the combined use of long-acting bronchodilators and inhaled corticosteroids to optimize outcomes in patients with inadequate control on monotherapy [1]. Both long-acting bronchodilators (including the anticholinergic tiotropium bromide [TIO] and long-acting beta-agonists [LABAs] such as salmeterol and formoterol) and combinations of LABAs and inhaled corticosteroids (ICS) (such as fluticasone-salmeterol [FSC] and budesonide-formoterol) have been shown to improve dyspnea, reduce exacerbation rates, and enhance health-related quality of life compared with placebo [2-8]. Furthermore, combination ICS/LABAs have been shown to reduce exacerbation rates more than either monotherapy component alone [9-11]. In addition, triple therapy, which adds an anticholinergic to ICS/LABA combinations, has been associated with greater improvements in lung function and quality of life and reduced rates of hospitalization compared with anticholinergic therapy alone [12-17]. For example, in a randomized, double-blind, placebo-controlled study of 449 patients with moderate or severe COPD, addition of FSC to TIO improved lung function and quality of life and reduced hospitalization rates, although it did not significantly reduce the rates of healthcare utilization-defined COPD exacerbations over a 1-year period [12]. The benefits of triple therapy over anticholinergic therapy alone have been attributed to maintenance of airway functioning through complementary mechanisms (ie, cholinergic receptor blockade and beta receptor activation) [13,16].

While the efficacy and tolerability of triple therapy have been evaluated in clinical trials, the effect on COPD outcomes of triple therapy relative to TIO alone has not been assessed in the real-world setting (ie, outside the confines of a controlled clinical trial). Furthermore, although research on the pharmacoeconomic impact of FSC and ipratropium has been done [18], the potential impact of triple therapy relative to TIO alone on healthcare costs has not been assessed. The study reported herein was conducted to compare the risks of COPD exacerbations and COPD-related healthcare costs between patients initiating TIO alone and patients initiating triple therapy with FSC added to TIO.

## Methods

### Study design

**Figure 1:**
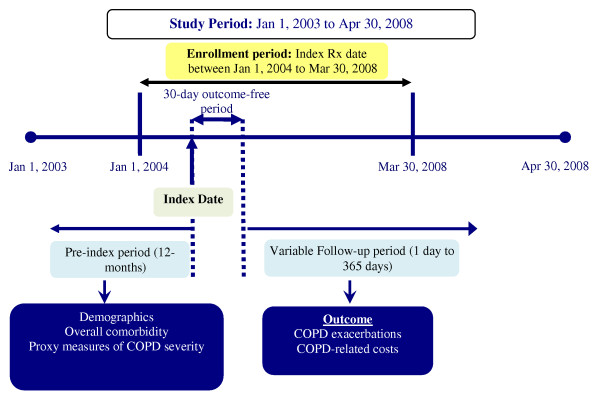
**Study design**.

Figure 1 shows the design of this observational, retrospective cohort study. The target population was managed care enrollees having ≥ 1 pharmacy claim for TIO during the study period, which extended from January 1, 2003, through April 30, 2008. An index TIO prescription was defined as the first chronologically occurring pharmacy claim for TIO during the enrollment period, which extended from January 1, 2004 through March 30, 2008. The date of the index TIO prescription was denoted the *index date*. The *pre-index period *was the 1-year period before the index date. The *post-index period *included a *30-day outcome-free period *(so named because study outcomes were not assessed during that period) and a *variable-length follow-up period *to a maximum of 1 year during which study outcomes were assessed. The 30-day outcome-free period was included to provide sufficient time for the new medication to take effect. The length of the follow-up period was defined for each patient based on the first occurrence of any of the following events: > 60-day gap between end of days supply of the preceding prescription and the fill date of the next consecutive prescription, an exacerbation (defined below) or COPD-related hospitalization or emergency room visit (defined below), end of continuous eligibility in the health plan, end of the study period, or the end of the 1-year follow-up period. This study was exempt from institutional review board approval as it was retrospective, did not involve an intervention, and utilized anonymized data.

### Data source

Data were obtained from the IMS LifeLink Health Plans Claims Database [19], an integrated source of fully adjudicated managed-care claims containing data from more than 90 managed healthcare plans covering more than 60 million lives across the United States. Data available for each patient include inpatient and outpatient diagnoses (by *International Classification of Diseases 9th Edition, Clinical Modification *[ICD-9-CM] diagnosis code) and procedures (in *Current Procedural Terminology, Version 4 *[CPT-4] and Healthcare Common Procedure Coding System [HCPCS] formats) and both retail and mail-order prescription records, which include the National Drug Code (NDC) code and quantity dispensed. Charged, allowed, and paid amounts are available for all services rendered as are dates of service for all claims. Additional data elements include demographic variables (age, gender, geographic region), payor type (Medicare, Medicaid, commercial, self-insured), provider specialty, and start and stop dates for plan enrollment. The data are fully de-identified and compliant with the Health Insurance Portability and Accountability Act (HIPAA).

### Sample

The target population was managed-care enrollees who had an index event of at least one pharmacy claim for TIO during the study period (January 1, 2003, through April 30, 2008). Additional inclusion criteria were age ≥ 40 years at the index date, ≥ 1 diagnosis for COPD (ICD-9-CM codes 490.xx, 491.xx, 492.xx, 496.xx) in any diagnosis field during the pre-index period or on the index date, ≥ 1 exacerbation (defined below) in the pre-index period, ≥ 1 prescription claim for ipratropium or ipratropium/albuterol combination in the pre-index period, ≥ 2 prescriptions for TIO (including the index prescription) during the post-index period, no prescription for FSC during the pre-index period, and no exacerbation or hospital/emergency room visit within 30 days after the index date. ICD-9-CM code 490.xx, bronchitis not specified as chronic or acute, was included in an attempt to enhance the validity of the analysis by capturing patients with chronic bronchitis whose condition was coded incorrectly. In addition, patients had to be continuously eligible to receive healthcare services in the pre-index period and for at least 30 days after the index date. The following conditions occurring in the pre- or post-index period were reasons for exclusion: respiratory cancer, cystic fibrosis, fibrosis due to tuberculosis, bronchiectasis, pneumonoconiosis, pulmonary fibrosis, pulmonary tuberculosis.

Patients were categorized on the index date into one of two cohorts (TIO+FSC or TIO) depending on their medication use. Patients in the TIO+FSC cohort had combination therapy with TIO and FSC, defined as having an FSC claim on the same date as the TIO claim. Patients in the TIO cohort had no FSC use. Patients adding FSC for the first time after the 30-day outcome-free period were excluded from the sample to ensure that the TIO cohort was not also using FSC. Patients assigned to a drug therapy cohort were considered to be using that therapy during the entire follow-up period regardless of whether they switched therapy to another maintenance medication (ie, LABA or ICS) (< 5% of the sample switched to a LABA or ICS in the follow-up). While FSC 250 μg is the currently approved daily dose for COPD in the United States, this study did not exclude from the sample patients using higher or lower doses of FSC.

### Outcomes

The risks of moderate COPD exacerbation and severe COPD exacerbation were compared between cohorts. Moderate COPD exacerbation was defined as an emergency room visit with a primary diagnosis code for COPD, a physician visit with a diagnosis code for COPD in any field + a prescription for an oral corticosteroid, a physician visit with a diagnosis code for COPD in any field + an antibiotic for respiratory infections, or physician administration of nebulized albuterol within 3 days following a physician office visit. Severe COPD exacerbation was defined as hospitalization with a primary discharge diagnosis for COPD. When computing the number of exacerbations, an exacerbation within 45 days of a previous exacerbation was not counted as a separate exacerbation [20]. Risks of emergency room visits with a primary diagnosis code for COPD and hospitalizations with a primary discharge diagnosis for COPD were also examined separately in case they were missed in the count of exacerbations (eg, if they occurred within 45 days of a previous exacerbation). COPD-related total (medical+pharmacy), medical, and pharmacy costs were also determined. COPD-related medical costs were computed from the paid amounts of medical claims with a primary diagnosis code for COPD. COPD-related pharmacy costs were computed from paid amounts of COPD-related prescription medications (including anticholinergics, short-acting beta agonists, LABAs, ICSs, combination ICS/LABAs, methylxanthines, oral corticosteroids, and antibiotics for respiratory infections) identified using NDC codes, HCPCS codes beginning with the letter J, or CPT codes as appropriate.

All study outcomes were assessed during a variable follow-up period as described above. Costs were computed on a per-month basis because of interpatient differences in the length of follow-up and were standardized to 2008 US dollars using the medical component of the Consumer Price Index.

Cohorts were also compared with respect to pre-treatment characteristics including demographics (age, sex, US census region), comorbidities during the pre-index-period (Charlson comorbidity index score [21], asthma), and proxies for COPD severity during the pre-index period (number of canisters of inhaled SABAs, number of canisters of inhaled ipratropium, number of prescriptions for oral corticosteroids, number of classes of maintenance therapy, use of home oxygen therapy, number of hospitalizations/emergency room visits for COPD, presence of an intensive care unit stay for COPD, number of physician visits for COPD). Age, sex, and US geographic region at the index date were obtained from enrollment files. The Dartmouth-Manitoba adaptation of the Charlson comorbidity index score [21]-a weighted index of 19 chronic medical conditions that predict mortality, post-operative complications, and length of hospital stay-was calculated for each patient based on diagnoses reported during the pre-index period. COPD codes that are generally included in the computing the Charlson index were excluded. Patients were classified as having asthma if they had ≥ 1 hospitalization or emergency room visit or ≥ 2 physician visits with a diagnosis of asthma in any field. The number of canisters of SABA was computed by dividing the quantity dispensed in mg by mg per canister. The use of home oxygen therapy was categorized as a binary variable (use, no use) based on CPT codes for home oxygen therapy on medical claims. The presence of an intensive care unit stay with a primary diagnosis code for COPD was identified via revenue codes.

### Statistical analysis

Pretreatment characteristics were summarized with descriptive statistics. Inferential statistics (chi-square test for categorical variables, t-test or Mann-Whitney test for continuous variables) were used to quantify differences between cohorts.

Unadjusted outcomes of COPD-related exacerbations assessed during the follow-up period were reported as rate per 100 person-years of follow-up. The log-rank test was used to assess statistical differences in unadjusted time to COPD-related exacerbations, and *P *values were used to evaluate statistical differences in the unadjusted rate per 100 person-years of follow-up. Survival analysis techniques were used to determine differences in time to COPD-related exacerbations after controlling for all other baseline covariates. The proportional hazards assumption was tested using the global test of proportional hazards to assess the appropriateness of a Cox-proportional hazards regression. Hazard ratios from survival analysis models provided an estimate of the adjusted differences in time to COPD-related exacerbations.

Unadjusted costs per month were summarized with descriptive statistics for all costs and cost components. Differences in unadjusted costs per month were analyzed using *t*-tests or Mann-Whitney tests for all costs and cost components. Differences between cohorts in adjusted COPD related total, medical, and pharmacy costs were assessed using multivariate regression models. A generalized linear model (GLM) using a gamma distribution with a log link was used. This model estimates the adjusted costs directly, without the need for retransformation, while simultaneously using log-transformed costs in its estimation. Because the data were characterized by a significant proportion with zero costs and a positively skewed distribution for those with costs, 2-part models were used with a logistic model for the first part and a GLM model for the second part. Adjusted costs estimated from 2-part models were computed by multiplying the adjusted probability obtained from the logistic regression model (part 1) with the predicted cost from the GLM model (part 2). Covariates included in the model for statistical adjustment included age, sex, US census region, Charlson comorbidity index score, asthma diagnosis, and proxies for COPD severity during the pre-index period (number of canisters of inhaled short-acting beta agonists, number of canisters of inhaled ipratropium, number of prescriptions for oral corticosteroids, number of classes of maintenance therapy, use of home oxygen therapy, number of hospitalizations/emergency room visits for COPD, number of physician visits for COPD, and number of exacerbations). Additionally, models with costs as the dependent variable included pre-index COPD-related costs.

## Results

### Sample

The number of persons in the database with a ≥ 1 prescription claim for TIO during the study period was 85,153 (Table 1). Of this group, 3333 patients (*n *= 852 FSC+TIO, *n *= 2481 TIO) met all eligibility criteria and comprised the study sample. The most common reason for exclusion of those with ≥ 1 prescription claim for TIO during the study period from the study sample was absence of a COPD-related exacerbation in the pre-index period (72.4%, or 61,619 of the patients excluded) (Table 1).

**Table 1:** Sample Derivation, Demographics, and Pre-Index Clinical Characteristics and Costs

Sample Derivation							
**Total number of patients with ≥ 1 TIO prescription during study period **							85,153
**Reason for exclusion, n (%)**							
< 40 years old as of index date							3347 (3.9)
Absence of continuous health plan eligibility							19,743 (23.2)
Absence of ≥ 1 claim with diagnosis code for COPD							29,216 (34.3)
Presence of FSC in the pre-index period							20,510 (24.1)
Presence of exclusionary comorbid conditions							14,448 (17.0)
Absence of index TIO prescription date during enrollment period							219 (0.26)
Absence of COPD-related exacerbation in the pre-index period							61,619 (72.4)
Absence of ipratropium/ipratropium-albuterol in the pre-index period							22,691 (26.7)
Presence of a COPD-related exacerbation or hospital/emergency room visit in 30-day outcome-free period							6317 (7.4)
Follow-up ending during the 30-day outcome-free period							10,779 (12.7)
**Final sample, N**							3333

	**Total**		**TIO**		**TIO+FSC**		***P*-value**
		
	N = 3333		*n *= 2481		*n *= 852		

**Demographics**							

Mean age, years (SD)	65.7	(10.9)	66.1	(10.9)	64.6	(11.0)	0.0008

Male, n (%)	1565	(47.0%)	1166	(47.0%)	399	(46.8%)	0.9332

Region, n (%)							
East	970	(29.1%)	711	(28.7%)	259	(30.4%)	
Midwest	1283	(38.5%)	938	(37.8%)	345	(40.5%)	
South	560	(16.8%)	404	(16.3%)	156	(18.3%)	
West	520	(15.6%)	428	(17.3%)	92	(10.8%)	

**Comorbidity in pre-index period**							

Charlson index (mean, SD)	1.98	(2.1)	1.91	(2.1)	2.18	(2.3)	0.0018

Presence of asthma (%)	1084	(32.5%)	757	(30.5%)	327	(38.4%)	< .0001

**COPD severity in pre-index period**							

Mean (SD) short-acting beta-agonist canisters	2.38	(5.1)	2.43	(5.0)	2.22	(5.4)	0.3197

Mean (SD) oral corticosteroid prescriptions	2.03	(2.8)	2.09	(2.9)	1.87	(2.3)	0.0253

Mean (SD) ipratropium or ipratropium/albuterol canisters	5.01	(6.9)	5.12	(6.9)	4.72	(6.9)	0.1451

Mean (SD) classes of maintenance medication*	0.48	(0.7)	0.52	(0.7)	0.37	(0.6)	< .0001

Use of home oxygen therapy, n (%)	1049	(31.5%)	801	(32.3%)	248	(29.1%)	0.0849

Presence of intensive care unit stay for COPD, n (%)	110	(3.3%)	81	(3.3%)	29	(3.4%)	0.8447

Hospital visits for COPD							

n (%)	695	(20.9%)	467	(18.8%)	228	(26.8%)	< .0001

Mean (SD)	0.24	(0.5%)	0.22	(0.5%)	0.31	(0.5%)	0.0002

Emergency room visits for COPD							

n (%)	769	(23.1%)	570	(23.0%)	199	(23.4%)	0.8193

Mean (SD)	0.30	(0.7%)	0.31	(0.7%)	0.29	(0.6%)	0.3304

Hospital or emergency room visits for COPD							

n, (%)	1289	(38.7%)	917	(37.0%)	372	(43.7%)	0.0005

Mean (SD)	0.55	(0.9)	0.53	(0.9)	0.59	(0.8)	0.0813

Mean (SD) number of physician visits for COPD	3.23	(3.6)	3.33	(3.6)	2.93	(3.4)	0.0041

**Exacerbations in pre-index period**							

All, mean (SD)	1.49	(0.8)	1.50	(0.8)	1.45	(0.7)	0.1441
							
Severe							
n (%)	535	(16.1%)	360	(14.5%)	175	(20.5%)	< .0001
Mean (SD)	0.18	(0.4)	0.16	(0.4)	0.22	(0.4)	0.0007

Moderate							

n (%)	3021	(90.6%)	2274	(91.7%)	747	(87.7%)	0.0006
Mean (SD)	1.31	(0.8)	1.34	(0.9)	1.23	(0.8)	0.0014

**Pre-index costs, mean (SD)**							

Total COPD-related costs	$9108	(18,434)	$8825	(18,786)	$9932	(17,355)	0.1164
COPD-related pharmacy costs	$1444	(1625)	$1494	(1681)	$1296	(1441)	0.0009
COPD-related medical costs	$7665	(18,220)	$7331	(18,536)	$8636	(17,242)	0.0618

*includes ipratropium, ICS, LABA

Table 1 shows demographics as well as pre-index clinical characteristics, healthcare use, and costs. The cohorts were generally comparable with some exceptions. The TIO+FSC cohort was slightly younger and had more comorbidities than the TIO cohort. During the pre-index period, severity measures related to medical use tended to reflect greater COPD severity in the TIO+FSC cohort whereas severity measures related to pharmacy use tended to reflect greater severity in the TIO cohort. For example, more patients in the TIO+FSC cohort than patients in the TIO cohort had a COPD-related hospitalization (26.8% versus 18.8%, *P *< 0.001) whereas the number of oral corticosteroid prescriptions was higher in the TIO cohort than the TIO+FSC cohort (2.09 versus 1.87, *P *= 0.025). These differences were reflected in the costs such that the TIO cohort had higher pharmacy costs than the TIO+FSC cohort ($1494 versus $1296, *P *< 0.001). Medical costs were higher for the TIO+FSC cohort than the TIO cohort, but the difference was not statistically significant.

### COPD-Related exacerbations

Patients in the TIO cohort had a longer follow-up time (in days) compared to the TIO+FSC cohort (mean [SD], median: 143.9 [118.4], 98.0 versus 106.7 [93.4], 66.5). Table 2 shows unadjusted and adjusted data on COPD-related exacerbations. After controlling for differences in baseline covariates between the cohorts, the TIO+FSC cohort had a 23% lower hazard of experiencing any COPD-related exacerbation (*P *= 0.007) and a 24% lower hazard of experiencing a moderate COPD-related exacerbation (*P *= 0.013) during the follow-up period. A reduction in the adjusted rate of combined hospitalization/emergency room visit with TIO+FSC versus TIO was also found but was not statistically significant.

**Table 2:** COPD-Related Adjusted Multivariate Outcomes and Unadjusted Outcomes in the Follow-up Period by Cohort

Adjusted Outcomes			
	Adjusted Hazard Ratio:** TIO+FSC vs TIO (reference cohort: TIO)**	**95% CI**	***P *value**

**Any exacerbation**	**0.772**	**(0.641, 0.930)**	**0.007**
Severe	0.622	(0.328, 1.180)	0.146
**Moderate**	**0.763**	**(0.646, 0.949)**	**0.013**

COPD-related hospital/emergency room visit	0.742	(0.511, 1.078)	0.118
Hospitalization	0.681	(0.400, 1.159)	0.157
Emergency room visit	0.807	(0.504, 1.293)	0.373

Unadjusted Outcomes: Rate per 100 Person-Years			

	TIO Alone	TIO+FSC	*P *value
	*n *= 2481	*n *= 852	

**Any exacerbation**	**95.58**	**75.38**	**0.003**
Severe	7.86	5.06	0.171
**Moderate**	**88.65**	**69.74**	**0.004**

COPD-related hospital/emergency room visit	21.29	15.79	0.070
Hospitalization	11.14	7.43	0.667
Emergency room visit	12.63	9.90	0.213

*P *values < 0.05 are bolded.

CI = confidence interval

### COPD-Related costs

Table 3 shows unadjusted and adjusted data on COPD-related costs. Unadjusted COPD-related total cost/month was significantly lower by ~$200 on average for the TIO+FSC cohort compared with the TIO cohort, a difference primarily attributable to a significant difference in medical cost/month. However, after adjusting for baseline differences, the lower monthly COPD-related medical cost/patient found for the TIO+FSC cohort compared with the TIO cohort ($490 versus $543, *P *> 0.05) was not statistically significant, and COPD-related total costs/month between cohorts were similar

**Table 3:** COPD-Related Adjusted Multivariate Costs (2008 USD) and Unadjusted Costs Per Month in the Follow-up Period by Cohort

	TIO	TIO + FSC	*P *value
	*n *= 2481	*n *= 852	
**Adjusted Costs: mean monthly costs per patient (95% CI)**			

Total	$721 ($190-$2005)	$721 ($149-$2250)	NS
Pharmacy	$190 ($67-$379)	$223 ($62-$490)	NS
Medical	$543 ($66-$3543)	$490 ($42-$4044)	NS

**Unadjusted Costs: mean (SD) monthly costs per patient**			

Total	$782 ($3496)	$598 ($1579)	0.039
Pharmacy	$194 ($220)	$206 ($195)	0.113
Medical	$588 ($3472)	$392 ($1554)	0.026

NS = nonsignificant

## Discussion

COPD exacerbations are associated with significant risk of persistent disability and death, incur substantial economic and clinical burdens in terms of healthcare resource use and costs, and are a major risk factor for subsequent serious exacerbations [1,22-27]. This study is, to the authors' knowledge, the first to assess risk of COPD exacerbations and COPD-related costs with triple therapy with an ICS/LABA and TIO compared with TIO monotherapy in the real-world setting as opposed to the setting of the controlled clinical trial. In this retrospective cohort study using claims data, initiation of triple therapy by adding FSC to TIO was associated with a 23% lower adjusted risk of experiencing any COPD-related exacerbation and a 24% lower adjusted risk of experiencing a moderate COPD-related exacerbation during the follow-up period after controlling for differences in baseline covariates between the cohorts. While a trend toward a benefit of triple therapy with FSC added to TIO over TIO monotherapy was observed in this study for severe exacerbations, it was not statistically significant. Consistent with the latter result, the risks of hospitalizations, emergency room visits, and combined hospitalizations and emergency room visits were lower with triple therapy with FSC added to TIO than with TIO monotherapy; however, these differences were not statistically significant. The risk analyses employed a rigorous approach to censoring variables to account for treatment discontinuation, loss of enrollment, and events of interest to ensure that outcomes were not inappropriately attributed to the treatment groups even after drug discontinuation.

The exacerbation data from this observational study extend the evidence base of the effect of triple therapy on exacerbation rates in COPD. While the beneficial effects of triple therapy versus TIO monotherapy on lung function have been consistently observed across studies [12-17,28], findings on the impact of triple therapy versus TIO monotherapy on exacerbation rates have been inconsistent [12,15,17]. In a recent 24-week, randomized, double-blind trial, no differences were found between triple therapy relative to TIO alone in the rate of healthcare utilization-defined exacerbations [17]. However, in a 1-year randomized, parallel-group study, triple therapy relative to TIO alone significantly reduced the number of healthcare utilization-defined, COPD-related, and all-cause hospitalizations, but did not significantly reduce the rate of exacerbations or the time to exacerbation [12]. Triple therapy relative to TIO alone decreased rates of severe exacerbations by 62% (p < 0.001) in a 12-week, randomized, double-blind, parallel-group study of budesonide formoterol [15]. Inconsistencies between clinical trials in effects of triple therapy versus TIO monotherapy on exacerbations have been linked to differences in the operational definitions of exacerbation [29]. In the current study, inclusive definitions for moderate exacerbation and, by extension, any exacerbation were employed. The definition of a moderate COPD exacerbation included not only emergency room visits, but also physician visits with a prescription for an oral corticosteroid or antibiotic for respiratory infections or physician administration of nebulized albuterol within 3 days of an office visit. A similarly inclusive definition was used in recent studies of COPD exacerbations [30-33]. This broad definition of COPD exacerbations provides a sensitive measure of treatment outcomes and reflects the spectrum of manifestations of COPD exacerbations in clinical practice. Severe exacerbations, on the other hand, were defined more narrowly in the current study as hospitalizations with a primary discharge diagnosis for COPD. In claims-based analysis, limited data are available to determine the severity of exacerbations. In this study, a restrictive definition of exacerbations was employed to improve accuracy at the risk of sensitivity.

The reduction in risk of event was not associated with reduced cost. Although a trend toward lower cost with triple therapy was observed, it was not statistically significant. Pharmacy costs, on the other hand, were higher for the TIO+FSC cohort, but were not significant either, and the lack of significance can partly be attributed to the lower refill rate for the TIO+FSC cohort compared to the TIO alone cohort (mean [SD]: 2.8 [2.6] versus 3.5 [3.3]).

The study is not without limitations. Our primary study aim was to evaluate triple therapy in the sequential manner recommended by GOLD guidelines (ie, addition of an inhaled corticosteroid-containing product to existing long-acting bronchodilator therapy [eg, addition of LABA/ICS to TIO therapy]). However, the alternate pathway to triple therapy (ie, initial LABA/ICS therapy for patients with severe COPD followed by subsequent addition of TIO) is also possible and is not as infrequent, as found in our initial data extraction. We found that 24.1% of the total number of TIO users (provided in Table 1 as presence of FSC in pre-index period) had LABA/ICS before their first-ever TIO prescription before any study criteria were applied. We chose to exclude these patients from our cohort of triple therapy users, however, as we felt that including the patients who add TIO versus those who added LABA/ICS in a combined triple therapy cohort would create a heterogeneous cohort that would include a mix of very severe patients and relatively less-severe COPD patients. This would have the undesirable effect of inadequate adjustment of potential confounders when comparing to a TIO alone cohort, given limitations of our data source (in terms of lack of clinical parameters). We acknowledge that this alternate pathway of triple therapy is important and deserves evaluation of triple therapy on its own, but we chose to exclude these patients to preserve internal validity of our study findings by creating comparable cohorts as much as possible. Future research may want to evaluate both types of triple therapy cohorts. Although analyses controlled for differences in baseline disease severity and other baseline characteristics in both adjusted cost models and risk models in the current study, such adjustment is necessarily imperfect and does not obviate the need for further assessment in cohorts balanced with respect to baseline characteristics. The possibility remains of residual confounding due to between-cohort differences in patient characteristics that were not controlled for in multivariate analysis.

Besides the possibility of residual confounding, other limitations of this study include potential errors in the coding of claims, the inability to verify the accuracy of diagnosis codes, and the absence of a means of assessing patient compliance. Furthermore, asthma, which is commonly comorbid with COPD and often treated similarly, could not be excluded based on diagnosis codes because of lack of reversibility information in claims data. The potential for these biases is inherent in observational studies. In the event that these biases were operating, they are likely to have been operating similarly between cohorts.

## Conclusions

In summary, results of this study show that triple therapy with FSC added to TIO compared with TIO monotherapy was associated with significant reductions in the adjusted risks of moderate exacerbation and any exacerbation over a follow-up period of up to 1 year and a nonsignificant reduction in COPD-related adjusted monthly medical costs. These data from the real-world setting, considered in the context of data from randomized clinical trials [12-17], provide preliminary support of the practice of utilizing triple therapy to optimize outcomes in COPD. The clinical data show that triple therapy has been associated with greater improvements in lung function and quality of life and reduced rates of hospitalization compared with TIO alone [12-14,17]. The results of the current analysis are consistent with the possibility that these improvements may be gained with triple therapy at roughly equal cost of that of TIO alone.

## Abbreviations

COPD: chronic obstructive pulmonary disease; CPT-4: *Current Procedural Terminology: Version 4; *FSC: fluticasone-salmeterol combination; GLM: generalized linear model; HIPAA: Health Insurance Portability and Accountability Act; ICD-9-CM: *International Classification of Diseases 9th Edition: Clinical Modification; *ICS: inhaled corticosteroids; LABA: long-acting beta-agonist; SABA: short-acting beta-agonist; TIO: tiotropium

## Competing interests

**Conflict of Interest Disclosure**: The authors acknowledge Jane Saiers, PhD (The WriteMedicine, Inc.) for assistance with preparing the manuscript. Dr. Saiers' work was funded by GlaxoSmithKline. Drs. D'Souza and Shah are employees of Xcenda, which received funding from GlaxoSmithKline for conducting this study. Mr. Bechtel, Dr. Dalal, and Dr. Crater are employees of GlaxoSmithKline and own company stock. Dr. Chatterjee has previously received compensation for speaking from GlaxoSmithKline, Boehringer Ingelheim, AstraZeneca, Novartis and Pfizer. He has been compensated for being a member of Scientific Advisory Boards for GlaxoSmithKline and Omnicare. He receives respiratory research funding from the National Institutes of Health, which did not fund this effort. He is a Reserve Component officer in the United States Navy. This work represents his opinion and not that of the US Navy or the Department of Defense.

## Authors' contributions

All authors participated in writing and review of the manuscript, and all authors approved the final version for submission to the journal. All authors 1) have made substantial contributions to conception and design, or acquisition of data, or analysis and interpretation of data; 2) have been involved in drafting the manuscript or revising it critically for important intellectual content; and 3) have given final approval of the version to be published.
